# MR-Guided Radiotherapy for Prostate Cancer

**DOI:** 10.3389/fonc.2020.616291

**Published:** 2020-12-09

**Authors:** Boris R. Tocco, Amar U. Kishan, Ting Martin Ma, Linda G. W. Kerkmeijer, Alison C. Tree

**Affiliations:** ^1^ Department of Radiotherapy, The Royal Marsden NHS Foundation Trust, London, United Kingdom; ^2^ University of California, Los Angeles, Los Angeles, CA, United States; ^3^ Radboud University Medical Center, Nijmegen, Netherlands; ^4^ Department of Radiotherapy and Imaging, Institute of Cancer Research, London, United Kingdom

**Keywords:** prostate cancer, MR-linac, image-guided radiotherapy, online adaptive radiotherapy, MR-guided radiotherapy

## Abstract

External beam radiotherapy remains the primary treatment modality for localized prostate cancer. The radiobiology of prostate carcinoma lends itself to hypofractionation, with recent studies showing good outcomes with shorter treatment schedules. However, the ability to accurately deliver hypofractionated treatment is limited by current image-guided techniques. Magnetic resonance imaging is the main diagnostic tool for localized prostate cancer and its use in the therapeutic setting offers anatomical information to improve organ delineation. MR-guided radiotherapy, with daily re-planning, has shown early promise in the accurate delivery of radiotherapy. In this article, we discuss the shortcomings of current image-guidance strategies and the potential benefits and limitations of MR-guided treatment for prostate cancer. We also recount present experiences of MR-linac workflow and the opportunities afforded by this technology.

## Introduction

Prostate cancer has accounted for 23.2% of all male cancer diagnoses in Europe in 2020 so far (1), a large proportion of whom will be treated with external beam radiotherapy (EBRT) for localized disease. EBRT offers patients non-invasive radical treatment and the move toward hypofractionation has allowed treatment schedules to be shortened. The low estimated α/β ratio of prostate cancer hypothesizes a benefit of hypofractionation, which has subsequently been evidenced in a number of trials (2–4) and transitioned into clinical practice guidelines across Europe (5–7) and America (8). Such results have encouraged clinicians to explore the boundaries of ultra-hypofractionation (UHF), testing 5 or 7 fraction schedules with promising oncological results (9–11).

Whilst the biology of prostate cancer may lend itself to hypofractionation, multiple obstacles remain in the pursuit of accurate dose delivery. Inter- and intra-fractional variability of target organ morphology and position as well as organ-at-risk (OAR) deformation limit the safety of dose escalation and hypofractionation with current image-guided radiotherapy (IGRT) techniques. The HYPO-RT-PC trial, comparing UHF for localized prostate cancer to conventional fractionation, reported significantly higher levels of patient-reported acute bowel and urinary toxicity with UHF (11), though late-term toxicity appeared equivalent regardless of treatment arm. However, these findings were not correlated in acute toxicity findings from the PACE-B trial in which the SBRT cohort reported similar levels of acute toxicity to the standard fractionation cohort (10). These differences may be due to radiotherapy technique, underlying the importance of optimizing dose delivery. Specifically, the radiation planning technique used for 80% of patients in the HYPO-RT-PC trial was three-dimensional conformal RT, rather than the more modern intensity-modulated RT, which has been associated with lower absolute rates of toxicity (11). Additionally, more generous planning margins were placed around the prostate to mitigate uncertainties due to prostate motion. Thus, the absolute rates of toxicity in the HYPO-RT-PC trial are likely higher than would be expected with modern treatment planning and delivery. Nonetheless, toxicity remains a possibility with all techniques and this remaining toxicity is likely determined not only by intrinsic radiosensitivity but also by doses delivered to critical adjacent organs.

The use of IGRT in prostate cancer is associated with improved biochemical control and lower rates of toxicity (12–14). MR-guided radiotherapy (MRgRT) brings IGRT to a higher level with improved soft tissue contrast and online adaptive planning allowing for greater accuracy of fraction delivery. MRgRT provides the opportunity to improve cancer outcomes while reducing treatment-related toxicity. Presently, there are two commercially available systems from which current experiences are drawn: Elekta Unity (Elekta AB, Stockholm, Sweden) which uses a 1.5 Tesla MRI machine, and Viewray MRIdian MR Linacs (Viewray Inc, Oakwood, OH) which uses a 0.35 Tesla MRI (15).

In this review, we will explore the shortcomings of current IGRT methods and the potential benefit and limitations of online adaptive MRgRT in prostate cancer. We will also describe current clinician experience of MR-guided workflow and the potential opportunities for future development and trials.

## Shortcomings of Current IGRT Strategies​ 

Current IGRT techniques include the use of cone-beam CT (CBCT) and implanted fiducial markers (FM), which may be used in conjunction; however, both have their limitations. CBCT alone has poor soft tissue resolution, limiting the accuracy of prostate-prostate matching (16). The use of radiopaque fiducials allows for rigid-registration but provides little to no information about organ deformation, seminal vesicle location, or bladder or rectal distension (17). The placement of fiducial markers is also an invasive procedure. Uncertainties in current IGRT strategies require larger planning margins to account for internal margin and set-up error, which can increase toxicity. Inter-fraction volumetric changes of the prostate gland have also been observed in moderate and profound hypofractionation schedules (18–20) and, with the move toward ultra-hypofractionation, direct visualization of the prostate serves to ensure dose coverage.

Any inter-fraction displacement necessitating contour repositioning is purely based on prostate matching and does not take into account the potential for differential movement of target organs such as seminal vesicles and pelvic lymph nodes (21, 22). Peng et al. analyzed 486 daily CT scans for 20 patients and found that in around 30% of fractions translational shifts were unable to adequately mitigate anatomical changes, indicating a need for online adaptive radiotherapy (ART) (23). While dosimetric coverage of the lymph node areas may be retained if bladder and rectal filling is pristinely maintained from fraction to fraction (24, 25), changes in anatomy could lead to overdosing of adjacent organs such as the small bowel. Furthermore, there is an increasing trend to dominant intraprostatic lesion boosts (26–28), which require additional accuracy in prostate matching adjustments on traditional kV planar or CBCT imaging.

Intra-fraction movement is an additional issue, which is sub-optimally mitigated by many current IGRT strategies. The prostate itself can move between image acquisition and beam on. Furthermore, bladder filling or rectal gas movement may influence target organ position by the order of a few millimeters, sufficient to affect CTV coverage. Both CT-based and MR-based analyses have demonstrated significant rates of intra-fractional motion. Calypso four-dimensional localization systems with the use of implanted electromagnetic markers showed prostate displacement of >3 mm 13.2% of the time during treatment (29). Similarly, three-dimensional cine MRI tracking of fiducials found prostate motion >2 mm in 43% scans by 5 min of treatment (30).

Any corrections to the field may be rendered inaccurate during beam on due to the aforementioned target position diversity (31–33), or otherwise clinicians must extend the planning margin to cover the expected excursion of prostate motion (34). A small number of non-MRgRT systems have intra-fraction motion solutions such as Cyberknife, which uses KV imaging tracking of fiducial seeds. During a fraction, which may take up to 45 min, fiducial seeds are tracked and adjustments to position can be made at 30–60 s intervals (35). However, systems for managing intra-fraction motion on the basis of fiducial markers require exposure to low doses of ionizing radiation.

## Potential for Benefit With MRgRT for Prostate Cancer

**Figure 1:**
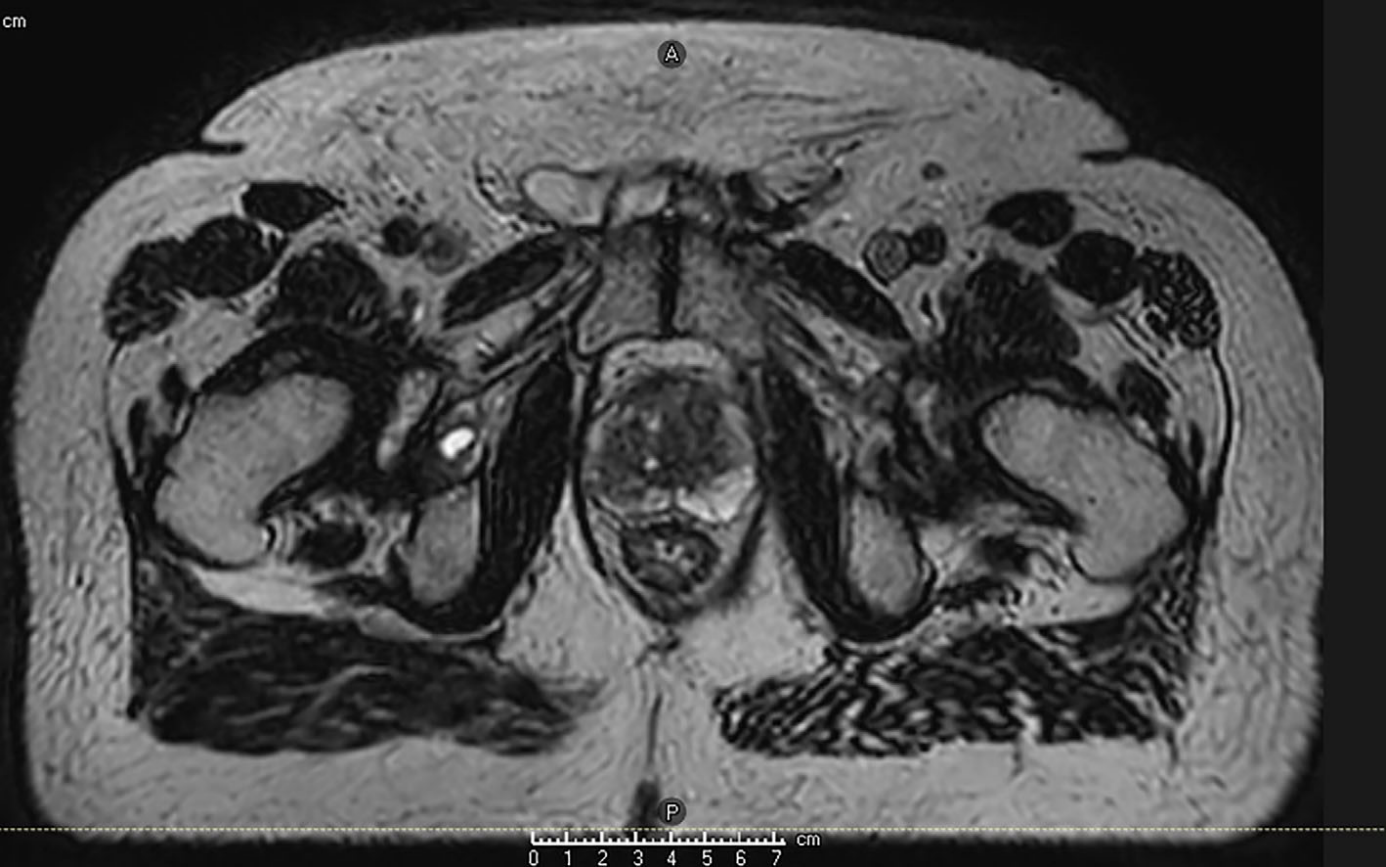
Axial image of the prostate (T2 2 min scan) from the Unity.

**Figure 2:**
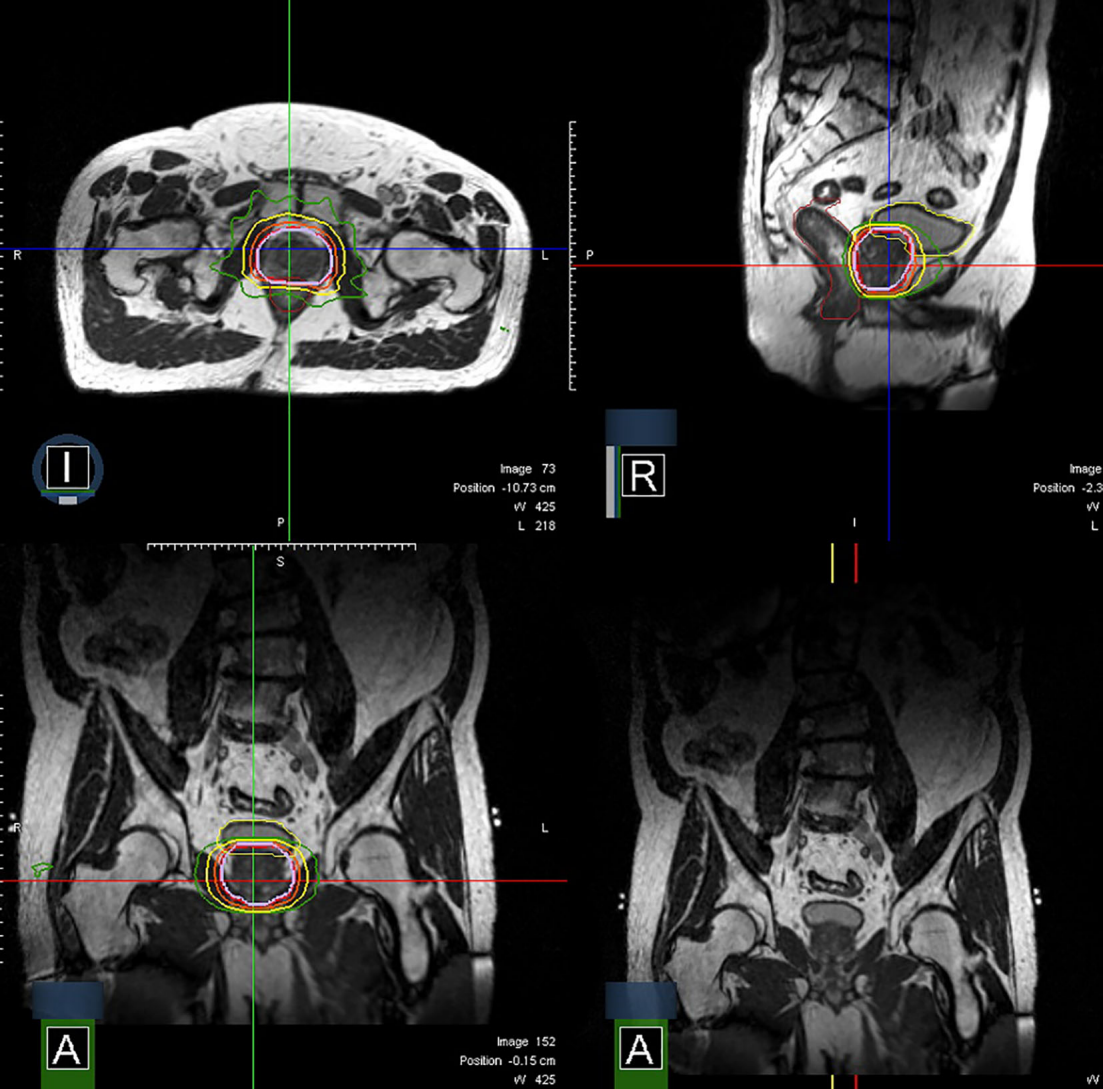
Axial, sagittal, and coronal images of a prostate plan on the MRIdian (isodoses: Red = 40 Gy, Orange = 36 Gy, Yellow = 24 Gy, Green = 20 Gy).

MRI guidance with or without ART has multiple potential advantages in terms of improving accurate dose delivery. First, because the prostate is much better visualized on MRI images compared to CT images, prostate CTVs generated by MRI are smaller and more precise than CT-based contours (36). **Figure 1** shows an image of the prostate from Unity. Typically, radiation-therapy planning MRIs are fused to CT simulation images to aid in contouring, but the fusion itself introduces 1–2 mm of residual error. Use of an MR-only workflow will bypass these issues. Second, on-board MRI imaging will allow direct tracking of the prostate, dispensing with the need for fiducials and sparing the patient an invasive procedure. Third, as a treatment course progresses, the daily image acquisition and adaptive re-planning allows for compensation related to prostate gland swelling, shrinkage, or deformation and inter-fractional motion of target or OARs. This daily sparing of OARs has the potential to decrease toxicity in both the short and long term. The ability to provide daily online adaptation minimizes inter-fraction uncertainty. **Figure 2** shows a daily adaptive prostate plan from a 0.35T MR-linac.

**Figure 3:**
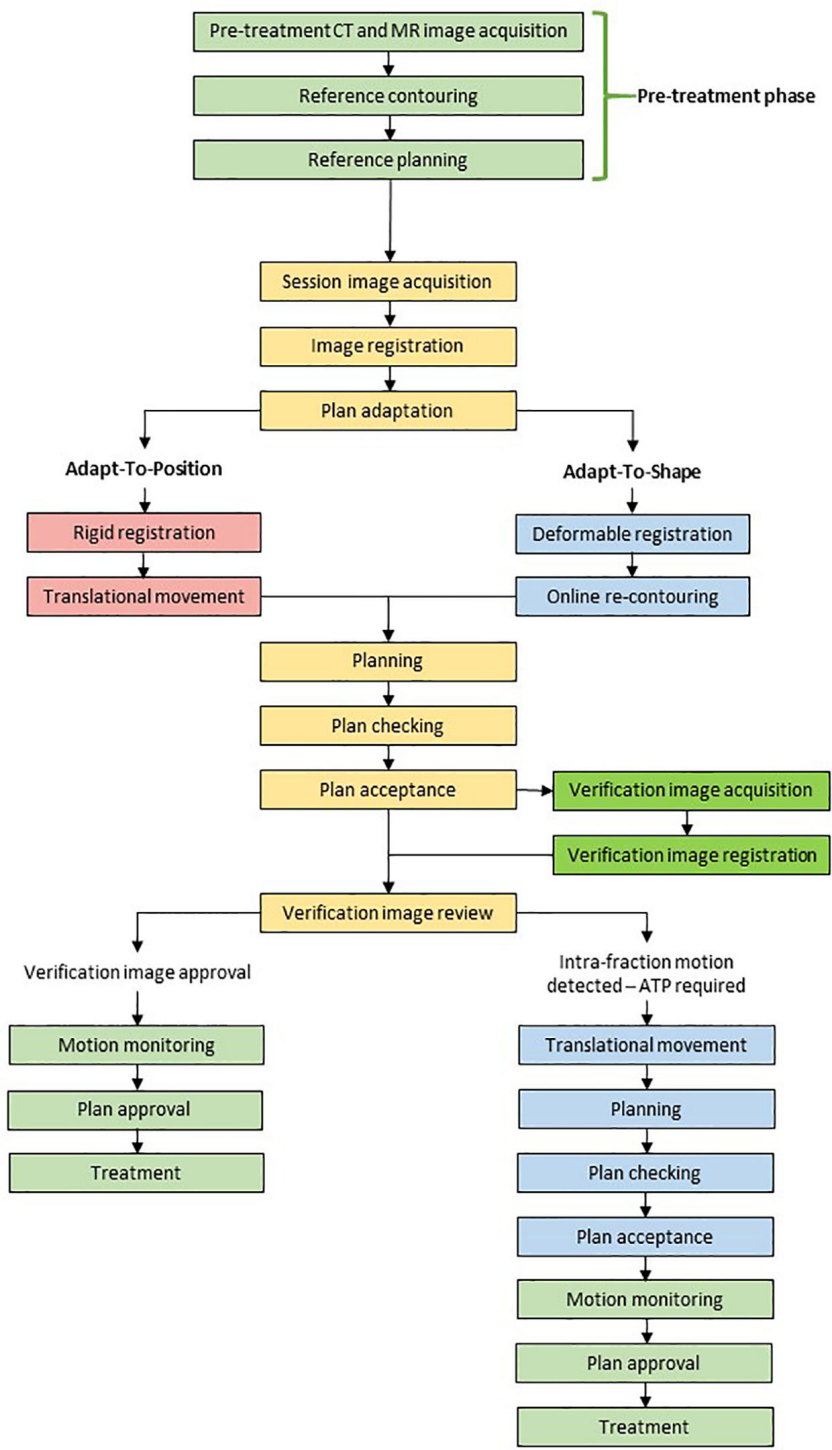
Example workflow for the Unity.

**Figure 4:**
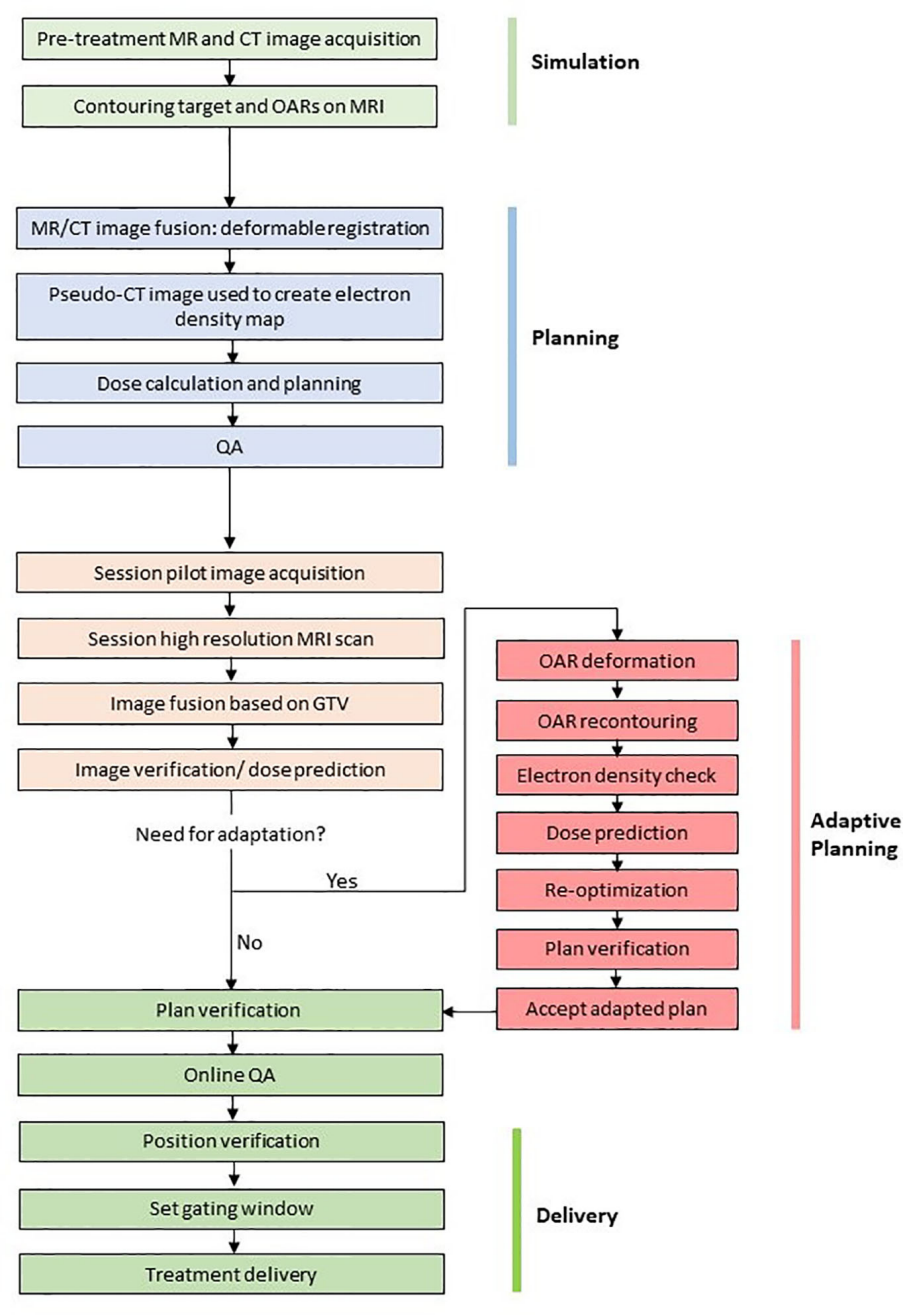
Example workflow for the MRIdian.

The workflow for the 1.5T MR-linac (Elekta Unity) is shown in **Figure 3** and the 0.35T MR-linac (Viewray MRIdian) in **Figure 4**. On the 0.35T MR-linac, a high resolution (1.5 mm isotropic voxel size or better) scan will be taken utilizing the on-board MRI to establish target and OAR geometry at the time of treatment. If deemed necessary, online ART with daily re-planning can be performed. During treatment, real-time imaging is acquired using MRIs obtained in a single sagittal plane at 4 frames per second, with a gating boundary on the prostate CTV at the physician’s discretion. Tolerances for the proportion of the CTV outside of the gating boundary can be set, and 2-dimensional table shifts can be performed as per the physician’s discretion.

For the 1.5T MR-linac, the decision to perform daily re-planning rests on review of daily anatomy alone. If anatomy has changed, re-contouring precedes a full re-optimization of the plan. The acquisition of a verification image subsequent to contouring and planning allows for there to be a shift of the new plan immediately prior to beam on (called ‘Adapt-to-Position’ workflow) to account for any prostate motion, which occurs during the workflow. Typically, this is due to rectal or bladder filling.

In the future, the prospect of intra-fraction dose adaptation brings us closer to the ideal online adaptive dose delivery system (37), capable of achieving the optimal balance of target dose and OAR sparing during the entirety of beam on.

## Current Experience of MRgRT in Prostate Cancer

Knowledge and experience of prostate MRgRT, on both Elekta Unity and Viewray MRIdian systems, has developed rapidly in the past few years. With MRgRT presenting a revolution in RT delivery, development of workflow and assessment of patient outcomes were initial priorities. Illustrative workflows are shown for the 1.5T MR-linac (**Figure 3**) and the 0.35T MR-linac systems (**Figure 4**). Such parameters were detailed by the Amsterdam VU team who described their experiences after 700 fractions were delivered (38).

MRgRT involves a multi-disciplinary team of radiographers, physicists, and clinicians. Most global experience is with daily re-contouring and re-planning. For example, the Amsterdam team reported that 97% of their delivered fractions were online ART plans (38).

The average duration of a delivered fraction is around 45 min, during, which time the patient is required to be on the treatment couch. The Amsterdam team also reported on a number of patient-reported outcomes and found that noise was the most common complaint (38, 39). Noise may be partially mitigated by the use of noise reduction headphones, which also enables communication between patients and radiation therapists during treatment (40). Our experience to date is that patients have not had any significant problems with the treatment, and patient experience is positive (41, 42). This is echoed by other practitioners including the group at VU University Medical Center (43).

Future studies about MR-linac clinical feasibility and patient toxicity outcomes are currently underway such as the Prostate Radiotherapy Integrated with Simultaneous MRI (PRISM study, NCT03658525), and the MOMENTUM study [The Multiple Outcome Evaluation of Radiotherapy Therapy Using the MR-linac Study (NCT04075305)] (44), which will help develop faster, more efficient workflows and benchmark multi-center patient outcomes. The ongoing Magnetic Resonance Imaging-Guided Stereotactic Body Radiotherapy for Prostate Cancer trial (MIRAGE trial, NCT04384770) is a phase III randomized study comparing standard CT-guided SBRT versus MRI-guided SBRT, with the primary endpoint of acute grade ≥2 genitourinary (GU) toxicity. It is designed as a superiority study, and secondary endpoints include patient-reported outcomes and late toxicity.

## Published Literature on Prostate MRgRT

Outcomes for prostate radiotherapy are expected to be good for most patients, with generally low levels of side effects and high expectations of efficacy. For these patients, the benefit of MRgRT will be hard to show. However, small or marginal gains will have a high population effect due to the number of prostate cancer patients and the high likelihood of cure. There is a subset of patients with challenging anatomy where inferior dose distributions have to be accepted to preserve OAR integrity. Dosimetric improvement over a course of 20 fractions has been shown, with the number of fractions achieving all target dosimetric goals being 86% for MRgRT and 80% for simulated conventional IGRT (45). For one patient with exceptionally challenging anatomy, the prostate CTV D98% delivered was 54.5 Gy with MRgRT and would have been 49.9 Gy with conventional techniques over 20 fractions. Therefore, even though reductions in bowel and bladder toxicity will be challenging to show on a population level, this technology could meaningfully impact quality of life in those who will live for many years after cure.

Small clinical series describing experiences with MRgRT for prostate cancer have been published previously and provided detailed suggestions about the proposed benefits, challenges, and future development in this cancer type (46–48). To date, only one prospective study has published outcomes. Bruynzeel et al. (39) published early toxicity results from a phase II study on MRg-SBRT for localized prostate cancer, which reported on RTOG and CTCAE clinician-reported and patient-reported outcomes (PROMs) for 101 patients for 3 months post-treatment with 36.25 Gy in 5 fractions. Clinician-reported outcomes suggested early GI and GU toxicity peaked at the final fraction of treatment and no grade 3 or higher toxicities were reported. The rates of grade ≥2 early GU and GI toxicities at the end of the treatment were 19.8% and 3%, respectively. The maximum cumulative grade ≥2 early GU and GI toxicity (by 12 weeks) measured by any symptom at any study time point was 23.8% and 5.0%. Patient-reported outcomes correlated closely with clinician reported outcomes with urinary toxicity peaking at the end of treatment and resolving by 3 months. The most common GI symptom was bloating. As a comparison to the above study, the PACE-B trial (10) showed a cumulative (exceeding baseline) CTCAE grade ≥2 GU and GI toxicity of 27.4% and 15.3% in the 5-fraction arm.

Tetar et al. (49) recently provided an update on the VU series with toxicity information extending through one year of follow-up. No grade 3 or higher toxicities were reported. All symptoms returned to baseline by 12 months. International prostate symptom scores (IPSS) returned to baseline 6 months post-treatment. 2.2% of patients reported GI symptoms at 1 year follow-up. Follow-up is too short to evaluate oncologic efficacy.

It is too early to form robust toxicity comparisons between MRgRT and non-MRgRT SBRT trials, but outcomes encourage further prospective and long-term trials to interrogate this important point.

## Limitations of MRgRT for Prostate Cancer

There are limitations to MRgRT for prostate cancer. The process of MRgRT provides a significant paradigm shift in the operation of radiotherapy departments, which necessitates updated safety training for staff, including all aspects of MR safety. Online ART requires the attention of several staff members for each treatment, often including a radiation oncologist, multiple radiographers and a physicist. Obviously, the person-hours required to deliver MRgRT are currently high when compared to traditional linac treatment, however efficiencies are likely to be forthcoming over time.

From a logistical point of view, there is limited availability of MR-linac machines and, as a result, clinician familiarity with such systems and online adaptive planning is still progressing. The predominance of radiation oncology experience until now has been centered on CT imaging and therefore the nuances and technicalities of MRI imaging are still being learned.

Maximum field size with 1.5T MR-linac machines could also lead to limitations of therapeutic capabilities and application in node-positive prostate cancer patients. With the current Unity maximum field size of 22cm in the superior-inferior (SI) plane, it is estimated that 80% of plans across cancer types would be suitable for MR-linac treatment (50) but a significant proportion of pelvic nodal irradiation fields would be too large. With the MRIdian Linac, the maximum field size is 24 cm SI, so a similar limitation applies. However, technical solutions to this limitation, and others, are being explored and current treatment possibilities do not represent the likely full capability of MR-linac machines (51). With prostate cancer predominantly affecting those over the age of 50, there is also likely to be a greater prevalence of medical contraindications to treatment on the MR-linac, thereby reducing numbers of suitable patients. From the patient perspective, the significantly longer time on the couch may deter some, and requires greater attention to patient comfort during treatment.

One element, which could reduce workload in the future of online ART, is automation of multiple components of the workflow. Auto-segmentation has been investigated and shown to decrease inter-observer variability while increasing dosimetric consistency on CT imaging (52, 53). This was replicated in MR-guided auto-delineation of pelvic organs although there has been evidence of poor concordance of auto-segmentation for targets such as seminal vesicles and the prostate (54–56). The creation of a library of contours and atlases from which an automated algorithm can learn will likely improve outcomes further. Currently, auto-generated contours are available for clinicians on both the 0.35T and 1.5T MR-linac machines and allow for a “warm-start contour” (i.e., not starting from scratch).

The duration of fraction delivery could also be aided by auto-segmentation. As mentioned, average duration of a single fraction for prostate cancer is around 45 min and this inevitably leads to greater bladder filling and variability of rectal distension, which have been shown to affect volume and position of the prostate and seminal vesicles to independent degrees (57). Current experiences are that intra-fraction OAR variation has not resulted in a significant number of adaptations required during beam on, although further published literature is required to confirm this. The role of auto-segmentation could reduce fraction duration and thereby minimize possible compromises to target organ dose delivery.

Another limitation of MRgRT is the risk of over-intervention with MRI imaging. The session MR image acquired at the beginning of each day’s treatment is a snapshot in time and one may devise a new plan based on that particular image with compromise of PTV coverage due to proximity of an OAR (e.g., bowel). OARs may move intra-fractionally (e.g., bowel peristalsis) and therefore may have unnecessarily compromised target coverage for that day.

## Opportunities for Future Development of MRgRT in Prostate Cancer 

While MRgRT provides hope for safe and effective dose delivery in prostate cancer treatment, further clinical studies are required to demonstrate a benefit.

Development of an MR-only, online workflow, without pre-treatment planning, would help to decrease radiotherapy pathway duration. Dispensing of the requirement for pre-treatment procedures, such as planning scans, would allow departments to condense pathways to benefit both clinicians and patients although acquisition of pre-treatment reference plans remains the standard in MR-only workflows currently (58). Removing the requirement to fuse planning CT to planning MRI would remove a potential source of error and uncertainty in the pathway. Although CT-based electron density calculations are considered to be the gold standard for radiotherapy planning, there are commercial MR-only solutions currently available, which may become more widely used (59).

Presently, operation of MRgRT requires a significant number of person-hours. Further streamlining of session times would be likely to result from incorporating auto-segmentation, as re-contouring is the most time-consuming component of the daily workflow. It remains to be seen if the accuracy of auto-delineation ever meets the standard set forth by radiation oncologists.

Amalgamation of roles within the inter-professional team may also reduce person-hours for treatment delivery. Inter-observer variation of MR contouring has shown good concordance (60) and is sure to lead to an evolution of roles within the MR-linac team starting with high volume, low complexity cases, which may become radiographer-led.

The predominant areas of opportunity lie within extreme hypofractionation in the online ART setting. Within the field of primary treatment of localized prostate cancer, ultra-hypofractionated SBRT schedules have been shown to be non-inferior to conventionally fractionated schedules (11). The increased levels of acute toxicity in the HYPO-RT-PC trial (11), and the lack of this in the PACE B trial (10), underline the importance of technical iteration to improve patient outcomes. Further studies to compare SBRT on traditional linac compared to MR-linac are under way, including the aforementioned phase III MIRAGE trial.

Many studies are undergoing to investigate possible superiority of dominant intraprostatic lesion (DIL) boosts (27). Doses of over 90 Gy equivalency have been shown to be safe (61, 62) but, as discussed above, our current IGRT strategies are imperfect for adapting to daily anatomical changes. Online ART using a 1.5T MR-linac would allow direct visualization of DILs during treatment. This is achievable on 1.5 T MR-linacs with diffusion scanning capabilities but, at present, 0.35T machines do not provide sufficient resolution to visualize DILs. Therefore rigid propagation is one option for this technique on a 0.35T MR-linac but alternative techniques to improve primary tumor visibility may be required; these have been employed in the diagnostic MR setting (63) but not as of yet in the therapeutic field. The feasibility of DIL visualization is also decreased with concomitant androgen deprivation therapy (62).

Other opportunities, beyond the scope of this review, include the use of MRgRT for post-operative prostatic bed irradiation or re-irradiation for radio-recurrent disease. The ability to provide more accurate dose-escalated treatment with direct visualization of tumor bulk has implications for post-prostatectomy relapses. The RADICALS-RT trial recently reported its 5 year results, which showed non-inferiority of salvage radiotherapy compared to adjuvant treatment (64). Currently, standard of practice is to treat the prostate bed empirically upon biochemical failure. The use of multi-parametric MRI (mpMRI) has been shown to be of use in detection of locally recurrent disease (65, 66); those with macroscopic disease on MRI could be triaged to treatment on the MR-linac with the possibility of macroscopic lesion boost (67, 68). In addition, as larger margins and a formulaic derivation of the target volume is currently used for prostate bed treatments, there is the prospect of reducing toxicity with MRgRT—one current phase II study promises to shed light on the efficacy and toxicity of MR-guided SBRT and CT-based SBRT delivered in the post-prostatectomy setting (NCT03541850). There are also few salvage treatments for locoregional recurrent disease after radical prostate EBRT. Early toxicity results of re-irradiation salvage SBRT are favourable (68–70) and further research into MR-guided salvage re-irradiation may be useful.

Qualitative and quantitative inter-fraction assessment of tumor response with functional MRI has implications for future treatment (71). The ability to directly visualize biological response to radiotherapy during a treatment course would allow the opportunity to tailor dose delivery. Online daily ART to target areas of persistent areas of restricted diffusion, for example, could possibly improve outcomes although implementation of functional imaging on MR-linac poses a number of challenges (72). For instance, there is a decrease in signal intensity of healthy prostate tissue on T2-weighted imaging during the course of treatment, which reduces visibility of the dominant intraprostatic lesion (73).

Thanks to the persistent and focused efforts of many prostate radiotherapy researchers over the last decade, significant GI and GU side effects of radiotherapy are becoming rarer. Effects of radiotherapy on sexual function are now the most prevalent long term side effect experienced by patients. The structures, which require dosimetric sparing in order to preserve sexual function, are not well elucidated, but it is thought that this is vascularly-mediated. Excellent outcomes have been seen after sparing the internal pudendal artery (74) using standard image-guidance strategies. As the vascular structures can be clearly seen on the MR-linac, it may be possible to preserve sexual function by sparing visualized vessels. Further study is planned.

As we progressively hypofractionate in prostate cancer, optimising image-guidance becomes ever-more important. Research is currently planned to investigate reducing the number of fractions below 5, to explore the limits of hypofractionation. The ONE SHOT trial aims to assess the efficacy of a 19 Gy fraction with 17Gy urethral sparing with a 2 mm margin. No grade 3 or higher GU and no grade 2 or higher GI toxicities were observed (75), although current HDR brachytherapy data suggests that a single fraction may be sub-optimal (76). Two-fraction HDR appears to have excellent outcomes and the MR-linac would be the perfect EBRT platform to test this in prostate cancer.

## Conclusions

MRgRT presents a new paradigm shift in the delivery of prostate radiotherapy. Increasing accuracy of delivery and promising early experience will further encourage larger investigations of the benefit of MRgRT. The use of MRgRT could abolish the requirement for pre-planning and lead to shorter pathways, potentially with improved outcomes. Cohort randomized trials are needed and these will require collaboration between industry and academic partners to provide robust evidence for practice.

## Author Contributions

BT composed the original draft and was responsible for incorporating alterations and produced a figure. AK, TM, and AT contributed figures and critically appraised the article. LK critically appraised the article. All authors contributed to the article and approved the submitted version.

## Funding

This work was supported by Cancer Research UK grant C33589/A28284.

## Conflict of Interest

AT receives institutional research funding from Elekta, Varian, and Accuray and has received honoraria/travel grants from Elekta. AK received research funding and honoraria from ViewRay, Inc., as well as honoraria and consulting fees from Varian Medical Systems, Inc. and consulting fees from Intelligent Automation, Inc.

The remaining authors declare that the research was conducted in the absence of any commercial or financial relationships that could be construed as a potential conflict of interest.
